# *catena*-Poly[(*E*)-4,4′-(ethene-1,2-di­yl)dipyridinium [[bis­(thio­cyanato-κ*N*)ferrate(II)]-di-μ-thio­cyanato-κ^2^ *N*:*S*;κ^2^ *S*:*N*]]

**DOI:** 10.1107/S160053681003624X

**Published:** 2010-09-15

**Authors:** Susanne Wöhlert, Mario Wriedt, Inke Jess, Christian Näther

**Affiliations:** a Institut für Anorganische Chemie, Christian-Albrechts-Universität Kiel, Max-Eyth-Strasse 2, 24098 Kiel, Germany

## Abstract

In the title compound, {(C_12_H_12_N_2_)[Fe(NCS)_4_]}_*n*_, each Fe^II^ cation is coordinated by four N-bonded and two S-bonded thio­cyanate anions in an octa­hedral coordination mode. The asymmetric unit consists of one Fe^II^ cation, located on a center of inversion, as well as one protonated (*E*)-4,4′-(ethene-1,2-di­yl)dipyridinium dication and two thio­cyanate anions in general positions. The crystal structure consists of Fe—(NCS)_2_—Fe chains extending along the *a* axis, in which two further thio­cyanate anions are only terminally bonded *via* nitro­gen. Non-coordinating (*E*)-4,4′-(ethene-1,2-di­yl)dipyrid­inium cations are found between the chains.

## Related literature

For general background, see: Wriedt & Näther (2009*a* ▶,*b* ▶); Wriedt *et al.* (2009*a* ▶,*b* ▶). For a description of the Cambridge Structural Database, see: Allen (2002 ▶).
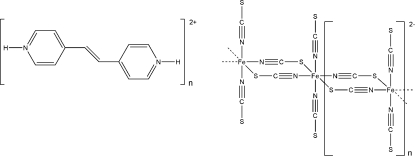


## Experimental

### 

#### Crystal data

(C_12_H_12_N_2_)[Fe(NCS)_4_]
*M* *_r_* = 472.41
Monoclinic, 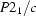

*a* = 5.7360 (2) Å
*b* = 11.5093 (4) Å
*c* = 15.0971 (6) Å
β = 96.562 (3)°
*V* = 990.14 (6) Å^3^
*Z* = 2
Mo *K*α radiation
μ = 1.20 mm^−1^
*T* = 293 K
0.16 × 0.13 × 0.09 mm

#### Data collection

Stoe IPDS-2 diffractometer
Absorption correction: numerical (*X-SHAPE* and *X-RED32*; Stoe & Cie, 2008 ▶) *T*_min_ = 0.826, *T*_max_ = 0.895
16607 measured reflections
2379 independent reflections
2173 reflections with *I* > 2σ(*I*)
*R*_int_ = 0.029

#### Refinement

*R*[*F* ^2^ > 2σ(*F* ^2^)] = 0.029
*wR*(*F* ^2^) = 0.068
*S* = 1.09
2379 reflections
124 parameters
H-atom parameters constrained
Δρ_max_ = 0.57 e Å^−3^
Δρ_min_ = −0.21 e Å^−3^

### 

Data collection: *X-AREA* (Stoe & Cie, 2008 ▶); cell refinement: *X-AREA*; data reduction: *X-AREA*; program(s) used to solve structure: *SHELXS97* (Sheldrick, 2008 ▶); program(s) used to refine structure: *SHELXL97* (Sheldrick, 2008 ▶); molecular graphics: *XP* in *SHELXTL* (Sheldrick, 2008 ▶) and *DIAMOND* (Brandenburg, 1999 ▶); software used to prepare material for publication: *XCIF* in *SHELXTL*.

## Supplementary Material

Crystal structure: contains datablocks I, global. DOI: 10.1107/S160053681003624X/im2228sup1.cif

Structure factors: contains datablocks I. DOI: 10.1107/S160053681003624X/im2228Isup2.hkl

Additional supplementary materials:  crystallographic information; 3D view; checkCIF report

## Figures and Tables

**Table:** 

Fe1—N11	2.1090 (16)
Fe1—N1	2.1165 (15)
Fe1—S1^i^	2.6375 (5)

**Table:** 

N11^ii^—Fe1—N1	89.61 (7)
N11^ii^—Fe1—N1^ii^	90.39 (7)
N11^ii^—Fe1—S1^i^	90.74 (5)
N1^ii^—Fe1—S1^i^	87.23 (4)
N11^ii^—Fe1—S1^iii^	89.26 (5)
N1^ii^—Fe1—S1^iii^	92.77 (4)

Symmetry codes: (i) 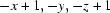; (ii)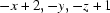; (iii) 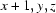.

**Fig. 1.**
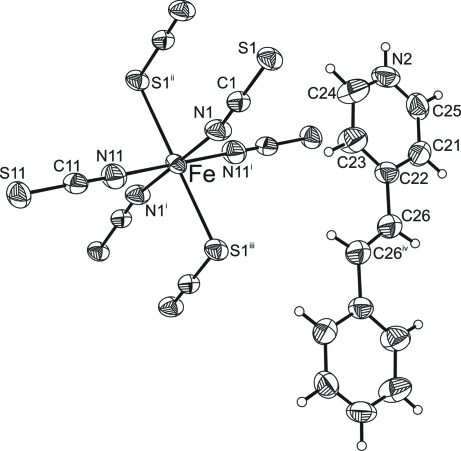
: Crystal structure of the title compound with labelling and displacement ellipsoids drawn at the 30% probability level. Symmetry codes: i = -x + 1, -y + 1, -z + 1; ii = -x+1, -y, -z+1; iii = +x+1, +y, +z; iv = -x+2, -y+1, -z+1.

**Fig. 2.**
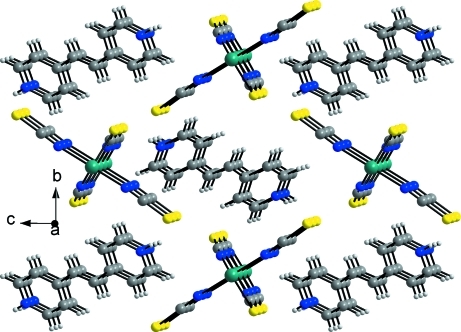
: Packing arrangement of the title compound with view approximately along the crystallographic a-axis.

**Fig. 3.**
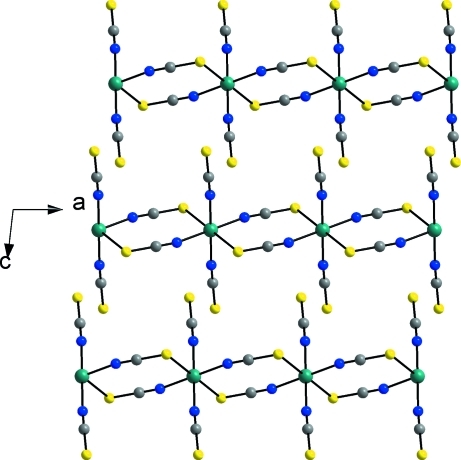
: Packing arrangement of the title compound with view on the inorganic part Fe(NCS)2–(NCS)2–Fe(NCS)2 approximately along the crystallographic b-axis. The non-coordinated organic cations were omitted for clearity.

## supplementary crystallographic information

### Comment

Recently, we have shown that thermal decomposition reactions are an elegant route for the discovering and preparation of new ligand-deficient coordination polymers with defined magnetic properties (Wriedt & Näther, 2009*a*, 2009*b*; Wriedt *et al.*, 2009*a*, 2009*b*). In our ongoing investigation on the synthesis, structures and properties of such compounds based on paramagnetic transition metal pseudo-halides and N-donor ligands, we have reacted iron(II) sulfate heptahydrate, potassium thiocyanate and *E*-1,2-di(4'-pyridyl)-ethene in water. In this reaction single crystals of the title compound were obtained, which were characterized by single crystal X-ray diffraction.

The title compound of composition [Fe(NCS)_4_]_n_-[*E*-1,2-di(4'-pyridinium)-ethene]_n_ (Fig. 1) represents an 1-D coordination polymer, in which each iron(II) cation is connected by four µ-1,3 bridging thiocyanato anions into chains that elongate in the direction of the crystallographic *a*-axis (Fig. 3). The octahedral coordination of each Fe cation is completed by two N-bonded thiocyanato anions. It must be noted that according to a search in the CCDC database (ConQuest Ver. 1.12 2010) such chains with transition metals are unknown (Allen, 2002).

Between the chains noncoordinating protonated (*E*)-4,4'-(ethene-1,2-diyl)dipyridinium cations are found, which are stacked in the direction of the crystallographic *a*-axis involving weak π-π-stacking interactions (Fig. 2). The FeN_4_S_2_ octahedron is slightly distorted with two long Fe—SCN distances of 2.6375 (5) Å and short Fe—NCS distances of 2.109 (2) and 2.116 (2) Å. The angles arround the metal atoms range between 87.23 (5) to 92.77 (5) and 180° (Tab. 1). The shortest intramolecular Fe···Fe distance amounts to 5.7360 (2) Å and the shortest intermolecular Fe···Fe distance amounts to 9.4919 (3) Å.

### Experimental

FeSO_4_ × 7 H_2_O and 1,2-di(4'-pyridyl)-ethene were obtained from Sigma Aldrich. KNCS was obtained from Alfa Aesar. 0.6 mmol (168.8 mg) FeSO_4_ × 7 H_2_O, 1.2 mmol (118.5 mg) KNCS and 0.15 mmol (28.2 mg) 1,2-di(4'-pyridyl)-ethene were reacted with 1 mL H_2_O in a closed test-tube at 120°C for three days. On cooling green block-shaped single crystals of the title compound were obtained in a mixture with unknown phases.

### Refinement

All H atoms were located in difference map but were positioned with idealized geometry and were refined isotropic with *U*_eq_(H) = 1.2 *U*_eq_(C,N) of the parent atom using a riding model with C—H = 0.93 Å and N—H = 0.86 Å.

### Crystal data

**Table:**

(C_12_H_12_N_2_)[Fe(NCS)_4_]	*F*(000) = 480
*M**_r_* = 472.41	*D*_x_ = 1.585 Mg m^−^^3^
Monoclinic, *P*2_1_/*c*	Mo *K*α radiation, λ = 0.71073 Å
Hall symbol: -P 2ybc	Cell parameters from 16607 reflections
*a* = 5.7360 (2) Å	θ = 2–28°
*b* = 11.5093 (4) Å	µ = 1.20 mm^−^^1^
*c* = 15.0971 (6) Å	*T* = 293 K
β = 96.562 (3)°	Block, green
*V* = 990.14 (6) Å^3^	0.16 × 0.13 × 0.09 mm
*Z* = 2	

### Data collection

**Table:**

Stoe IPDS-2 diffractometer	2379 independent reflections
Radiation source: fine-focus sealed tube	2173 reflections with *I* > 2σ(*I*)
graphite	*R*_int_ = 0.029
ω scans	θ_max_ = 28.0°, θ_min_ = 2.2°
Absorption correction: numerical (*X-SHAPE* and *X-RED32*; Stoe & Cie, 2008)	*h* = −7→7
*T*_min_ = 0.826, *T*_max_ = 0.895	*k* = −15→15
16607 measured reflections	*l* = −19→19

### Refinement

**Table:**

Refinement on *F*^2^	Primary atom site location: structure-invariant direct methods
Least-squares matrix: full	Secondary atom site location: difference Fourier map
*R*[*F*^2^ > 2σ(*F*^2^)] = 0.029	Hydrogen site location: inferred from neighbouring sites
*wR*(*F*^2^) = 0.068	H-atom parameters constrained
*S* = 1.09	*w* = 1/[σ^2^(*F*_o_^2^) + (0.0291*P*)^2^ + 0.4228*P*] where *P* = (*F*_o_^2^ + 2*F*_c_^2^)/3
2379 reflections	(Δ/σ)_max_ = 0.001
124 parameters	Δρ_max_ = 0.57 e Å^−^^3^
0 restraints	Δρ_min_ = −0.21 e Å^−^^3^

### Special details

**Table:**

Geometry. All esds (except the esd in the dihedral angle between two l.s. planes) are estimated using the full covariance matrix. The cell esds are taken into account individually in the estimation of esds in distances, angles and torsion angles; correlations between esds in cell parameters are only used when they are defined by crystal symmetry. An approximate (isotropic) treatment of cell esds is used for estimating esds involving l.s. planes.
Refinement. Refinement of F^2^ against ALL reflections. The weighted R-factor wR and goodness of fit S are based on F^2^, conventional R-factors R are based on F, with F set to zero for negative F^2^. The threshold expression of F^2^ > 2sigma(F^2^) is used only for calculating R-factors(gt) etc. and is not relevant to the choice of reflections for refinement. R-factors based on F^2^ are statistically about twice as large as those based on F, and R- factors based on ALL data will be even larger.

### Fractional atomic coordinates and isotropic or equivalent isotropic displacement parameters (Å^2^)

**Table:**

	*x*	*y*	*z*	*U*_iso_*/*U*_eq_	
Fe1	1.0000	0.0000	0.5000	0.03128 (10)	
N2	0.3720 (3)	0.38242 (17)	0.30355 (13)	0.0541 (4)	
H2	0.2544	0.3548	0.2697	0.065*	
S1	0.26743 (7)	0.16763 (4)	0.57486 (3)	0.03779 (11)	
C1	0.5284 (3)	0.12116 (14)	0.55629 (11)	0.0334 (3)	
N1	0.7106 (3)	0.08926 (14)	0.54301 (11)	0.0430 (4)	
S11	1.12064 (9)	−0.23939 (5)	0.76873 (3)	0.04587 (13)	
C11	1.0730 (3)	−0.15568 (16)	0.68107 (12)	0.0371 (4)	
N11	1.0414 (3)	−0.09600 (16)	0.61976 (11)	0.0490 (4)	
C21	0.6655 (4)	0.52070 (17)	0.32949 (15)	0.0522 (5)	
H21	0.7402	0.5868	0.3113	0.063*	
C22	0.7421 (3)	0.46922 (17)	0.41067 (13)	0.0425 (4)	
C23	0.6230 (4)	0.3727 (2)	0.43548 (15)	0.0571 (6)	
H23	0.6699	0.3361	0.4895	0.069*	
C24	0.4368 (4)	0.3310 (2)	0.38090 (17)	0.0620 (6)	
H24	0.3550	0.2664	0.3979	0.074*	
C25	0.4812 (4)	0.47453 (19)	0.27648 (15)	0.0555 (6)	
H25	0.4323	0.5077	0.2213	0.067*	
C26	0.9479 (4)	0.52118 (18)	0.46271 (14)	0.0494 (5)	
H26	1.0085	0.5891	0.4410	0.059*	

### Atomic displacement parameters (Å^2^)

**Table:**

	*U*^11^	*U*^22^	*U*^33^	*U*^12^	*U*^13^	*U*^23^
Fe1	0.02351 (16)	0.04009 (18)	0.03042 (17)	0.00428 (13)	0.00389 (12)	0.00449 (13)
N2	0.0395 (9)	0.0612 (11)	0.0580 (11)	−0.0001 (8)	−0.0097 (8)	−0.0187 (9)
S1	0.0277 (2)	0.0431 (2)	0.0430 (2)	0.00552 (16)	0.00590 (16)	−0.00552 (18)
C1	0.0305 (8)	0.0355 (8)	0.0338 (8)	−0.0023 (6)	0.0025 (6)	−0.0036 (6)
N1	0.0276 (7)	0.0470 (8)	0.0549 (9)	0.0011 (6)	0.0061 (6)	−0.0103 (7)
S11	0.0449 (3)	0.0521 (3)	0.0399 (2)	0.0004 (2)	0.00183 (19)	0.0133 (2)
C11	0.0297 (8)	0.0441 (9)	0.0372 (9)	−0.0029 (7)	0.0025 (6)	0.0003 (7)
N11	0.0494 (9)	0.0565 (10)	0.0395 (8)	−0.0047 (8)	−0.0020 (7)	0.0121 (7)
C21	0.0602 (13)	0.0386 (10)	0.0549 (12)	−0.0038 (9)	−0.0060 (10)	0.0019 (8)
C22	0.0389 (9)	0.0434 (10)	0.0435 (10)	−0.0004 (7)	−0.0029 (8)	−0.0071 (8)
C23	0.0644 (14)	0.0628 (13)	0.0417 (10)	−0.0140 (11)	−0.0043 (10)	0.0063 (9)
C24	0.0612 (14)	0.0671 (14)	0.0573 (13)	−0.0247 (12)	0.0048 (11)	−0.0017 (11)
C25	0.0639 (14)	0.0466 (11)	0.0510 (12)	0.0118 (10)	−0.0146 (10)	−0.0034 (9)
C26	0.0510 (11)	0.0445 (10)	0.0504 (11)	−0.0050 (8)	−0.0048 (9)	0.0022 (8)

### Geometric parameters (Å, °)

**Table:**

Fe1—N11	2.1090 (16)	C11—N11	1.150 (2)
Fe1—N11^i^	2.1090 (16)	C21—C25	1.359 (3)
Fe1—N1	2.1165 (15)	C21—C22	1.387 (3)
Fe1—N1^i^	2.1165 (15)	C21—H21	0.9300
Fe1—S1^ii^	2.6375 (5)	C22—C23	1.378 (3)
Fe1—S1^iii^	2.6375 (5)	C22—C26	1.469 (3)
N2—C25	1.320 (3)	C23—C24	1.360 (3)
N2—C24	1.324 (3)	C23—H23	0.9300
N2—H2	0.8600	C24—H24	0.9300
S1—C1	1.6437 (17)	C25—H25	0.9300
S1—Fe1^iv^	2.6375 (5)	C26—C26^v^	1.307 (4)
C1—N1	1.147 (2)	C26—H26	0.9300
S11—C11	1.6345 (19)		
			
N11—Fe1—N11^i^	180.0	N11—C11—S11	179.25 (19)
N11—Fe1—N1	90.39 (7)	C11—N11—Fe1	174.08 (17)
N11^i^—Fe1—N1	89.61 (7)	C25—C21—C22	120.0 (2)
N11—Fe1—N1^i^	89.61 (7)	C25—C21—H21	120.0
N11^i^—Fe1—N1^i^	90.39 (7)	C22—C21—H21	120.0
N1—Fe1—N1^i^	180.0 (9)	C23—C22—C21	117.88 (18)
N11—Fe1—S1^ii^	89.26 (5)	C23—C22—C26	125.17 (19)
N11^i^—Fe1—S1^ii^	90.74 (5)	C21—C22—C26	116.93 (19)
N1—Fe1—S1^ii^	92.77 (4)	C24—C23—C22	120.0 (2)
N1^i^—Fe1—S1^ii^	87.23 (4)	C24—C23—H23	120.0
N11—Fe1—S1^iii^	90.74 (5)	C22—C23—H23	120.0
N11^i^—Fe1—S1^iii^	89.26 (5)	N2—C24—C23	119.9 (2)
N1—Fe1—S1^iii^	87.23 (4)	N2—C24—H24	120.1
N1^i^—Fe1—S1^iii^	92.77 (4)	C23—C24—H24	120.1
S1^ii^—Fe1—S1^iii^	180.0	N2—C25—C21	119.8 (2)
C25—N2—C24	122.48 (18)	N2—C25—H25	120.1
C25—N2—H2	118.8	C21—C25—H25	120.1
C24—N2—H2	118.8	C26^v^—C26—C22	124.7 (3)
C1—S1—Fe1^iv^	100.68 (6)	C26^v^—C26—H26	117.6
N1—C1—S1	179.60 (19)	C22—C26—H26	117.6
C1—N1—Fe1	166.29 (15)		

Symmetry codes: (i) −*x*+2, −*y*, −*z*+1; (ii) −*x*+1, −*y*, −*z*+1; (iii) *x*+1, *y*, *z*; (iv) *x*−1, *y*, *z*; (v) −*x*+2, −*y*+1, −*z*+1.
